# Association of angiogenic factors (placental growth factor and soluble FMS-like tyrosine kinase-1) in preeclamptic women of African ancestry comorbid with HIV infection

**DOI:** 10.1007/s00404-024-07590-3

**Published:** 2024-06-24

**Authors:** Zinhle P. Mlambo, Motshedisi Sebitloane, Thajasvarie Naicker

**Affiliations:** 1 Optics and Imaging Centre, Doris Duke Medical Research Institute, Nelson R. Mandela School of Medicine, University of KwaZulu-Natal, Durban, South Africa; 2 Department of Obstetrics and Gynaecology, Nelson R. Mandela School of Medicine, University of KwaZulu-Natal, Durban, South Africa

**Keywords:** Preeclampsia, Placental growth factor, Soluble FMS-like tyrosine kinase 1, Vascular endothelial growth factor, HIV, Single nucleotide polymorphisms

## Abstract

**Background:**

Preeclampsia is a significant cause of maternal and fetal morbidity and mortality, particularly in low- and middle-income countries like South Africa.

**Aim:**

The aim of our study was to investigate the association between placental growth factor (PlGF) and soluble FMS-like tyrosine kinase-1 (sFlt-1) in South African preeclamptic women of African ancestry, comorbid with HIV infection.

**Methods:**

The study population consisted of women attending a regional hospital in Durban, South Africa, stratified by pregnancy type (normotensive pregnant and preeclampsia) and HIV status. Preeclampsia was defined as new-onset hypertension and proteinuria. DNA was obtained from whole blood. The SNPs of interest were rs722503 in sFlt-1 and rs4903273 in PlGF.

**Results:**

Our findings suggest that single nucleotide polymorphisms of rs722503 analysis show no significant associations between the genotypic frequencies of rs722503 variants and preeclampsia risk in either HIV-negative or HIV-positive groups of women of African ancestry. Similarly, the rs493273 polymorphism showed no significant association with preeclampsia risk in either HIV-negative or HIV-positive pregnant women. Additionally, comparisons of dominant, recessive, and over-dominant allele models did not reveal significant associations. These findings suggest that these genetic variants may not significantly contribute to preeclampsia development in this African ancestry population. However, significant differences were observed in the rs4903273 genotype frequencies between normotensive and preeclamptic women, regardless of HIV status, over dominant alleles AA + GG *vs* AG showed a significant difference [OR = 2.706; 95% Cl (1.199–5.979); adjusted *p* = 0.0234*], also in normotensive compared to EOPE (OR = 2.804; 95% Cl (1.151–6.89) *p* = 0.0326* and LOPE (OR = 2.601; 95% Cl (1.0310–6.539) *p* = 0.0492*), suggesting that they may be the potential role of this variant in preeclampsia susceptibility.

**Conclusion:**

The findings suggest that the rs722503 and rs493273 polymorphisms do not significantly contribute to preeclampsia susceptibility in HIV-negative or HIV-positive pregnant women. However, the rs4903273 genotype frequencies showed notable differences between normotensive and preeclamptic women, indicating a potential association with preeclampsia development in the African ancestry population irrespective of HIV status.

## What does this study add to the clinical work

**Table:** 

This study highlights the importance of monitoring angiogenic factors, particularly PlGF and sFlt-1, in preeclamptic women with HIV infection. It reveals significant insights into their potential role in the pathogenesis of preeclampsia, guiding more specific and effective management strategies for this high-risk group.	

## Introduction

Worldwide, an overwhelming 37.9 million individuals are infected by the Human Immunodeficiency Virus (HIV), hence this is a major health and economic challenge particularly in low- and middle-income countries (LMIC) such as South Africa (SA) [1]. The general HIV prevalence rate in SA is 13.9%, with a quarter of women aged being infected [1]. In pregnant women, the prevalence among 15–19 years and 20–24 years is 47.6% and 59.4% respectively, compared to older women of 35–49 years: 85.1%, *p* < 0.01) [2].

Additionally, SA faces challenges in reducing maternal and neonatal deaths [3]. Preeclampsia and other hypertensive disorders of pregnancy are the 3rd leading cause of maternal deaths and remain a major obstetric and public health concern, particularly in developing countries such as SA [3–6]. Preeclampsia is defined as a multisystem hypertensive disorder of pregnancy that affects 5–8% of all pregnancies worldwide [7, 8]. While the underlying pathophysiology of preeclampsia remains poorly understood, it is well-established that preeclampsia arises from a complex interaction between genetic, environmental, and immune factors [9, 10].

It is controversial whether HIV infection predisposes to preeclampsia development [11]. In an analysis of women who died from hypertensive diseases of pregnancy, women with HIV infection receiving High-active antiretroviral therapy (HAART) were at higher risk compared to non-infected women, [12, 13]. While the exact mechanisms underlying this association are unknown, increased expression of inflammatory cytokines, decreased placental perfusion, and endothelial dysfunction have been implicated [14, 15]. Moreover, since both conditions of preeclampsia and HIV infection affect angiogenesis, further research into the synergy of this comorbidity is urgent.

Angiogenesis is the growth of blood vessels from the existing vasculature [16]. Placental growth factor (PlGF) and soluble FMS-like tyrosine kinase 1 (sFlt-1), are two important angiogenic factors involved in the regulation of blood vessel growth during pregnancy [17–19]. In preeclampsia, the scavenging action of sFlt-1 reduces the bioavailability of VEGF [7, 20–22]. The measurement of PlGF and soluble FMS-like tyrosine kinase 1 (sFlt-1), has emerged as potential diagnostic and prognostic tools for preeclampsia development [23, 24]. Notably, PlGF is produced by the placenta and promotes angiogenesis and vascular expansion during pregnancy whilst sFlt-1 is anti-angiogenic and acts as a decoy receptor to bind VEGF (VEGF) and PlGF thereby decreasing their bioavailability [25, 26]. Several studies have demonstrated that sFlt-1 levels increase, in contrast to a decline in PlGF in preeclampsia [20, 27–30]. It is important to examine whether polymorphisms of these angiogenic factors occur in populations predisposed to preeclampsia. Single Nucleotide Polymorphisms (SNPs) are the most common form of genetic variation among individuals and can be found throughout the genome [31]. SNPs are an essential tool in genetics research, providing valuable information for understanding human genetic variation, disease risk, individual traits, and population dynamics [32].

Therefore, this study aims to evaluate the impact of single nucleotide polymorphisms (SNPs) (rs722503 and rs4903273) in sFlt-1 and PlGF of preeclampsia women of African ancestry comorbid with HIV infection.

## Materials and methods

### Study population and design

The DNA was extracted from whole blood samples obtained from pregnant women of African ancestry (n = 187). The primary study consisted of women attending a regional hospital in Durban, South Africa. Informed consent for the storage and future studies had previously been obtained from the Ethical Biomedical Committee at the University of KwaZulu-Natal (BCA040/12; BCA 3387/17). Study groups included normotensive pregnant women and preeclampsia women stratified by HIV status. Preeclampsia was defined as new-onset hypertension (blood pressure of ≥ 140/90 mmHg taken on two occasions, 4 h apart and at least 1 + proteinuria measured by urinary dipstick) commencing at 20 weeks or more of gestation, with or without proteinuria and/or with evidence of liver disease, renal complications, thrombocytopenia, and neurological dysfunctions [3]. The preeclampsia group was subdivided by gestational age into early-onset preeclampsia (EOPE), which refers to hypertension that occurs at < 33 weeks + 6 days of pregnancy, and late-onset preeclampsia (LOPE), which is hypertension that occurs at > 34 weeks of pregnancy [3]. All blood pressure and proteinuria measurements were recorded at the time of admission to the hospital or recorded at the time of recruitment for the normotensives.

#### DNA extraction and genotyping

DNA was isolated from 200 µl of whole blood using the QIA®amp DNA Blood Mini Kit, as per the manufacturer’s instructions (QIAGEN, Valencia, CA). Following extraction, DNA was eluded with nuclease-free water and stored at − 20 °C until genotyping analysis.

### TaqMan genotyping of PlGF and sFlt-1 gene polymorphisms

Two SNPs (rs722503, and rs4903273) were amplified to detect specific polymorphisms from purified DNA samples using a TaqMan master mix (Applied Biosystems by ThermoFisher Scientific) following the manufacturer’s protocol. Genotyping of SNPs was performed using the Quant Studio 7 real-time Flex PCR. The final reaction master mix contained a 20X working stock of TaqMan SNP genotyping assay (0.25 µl), 2X TaqMan universal master mix (2.5 µl), and DNA (3 µl), with a total volume of 5.75 µl per well of the master mix was added into each well of a MicroAmp Optical 96-well reaction plate (Applied Biosystems, Foster City, CA, USA). The TaqMan genotyping assay includes two fluorescently labeled primers for discriminating between two alleles of a specific SNP. On the 5′ ends, one primer is labeled with VIC® dye (a green fluorophore) for the wild-type allele and the other with 6-carboxyfluorescein (6-FAM™) dye (a blue fluorophore) for the mutant allele; the primer also contains a minor groove binder (MGB) and a non-fluorescent quencher on the 3′ ends. Following PCR amplification using the QuantStudio 5 Real-time PCR system (Applied Biosystems by ThermoFisher Scientific), allelic discrimination results were analyzed using QuantStudio™ design and analysis software version 1.5.2. An Excel database was created to compare SNP genotypes by pregnancy type (PE vs N; EOPE vs LOPE), HIV status as well as across study groups.

### Sample size

The sample size was determined using the Cohen’s effect minimum sample size with the statistical power of 80%; effect size = 0.40; type 1 (α) error = 0.05 was used. The study population consisted of 187 pregnant women; PE (n = 123) and normotensive pregnant (n = 64) women. The PE group was subdivided into EOPE (n = 64) and LOPE (n = 59) and was further stratified by HIV status EOPE− (n = 32), EOPE + (n = 30), LOPE− (n = 30), and LOPE + (n = 29). The normotensive group was equally divided by HIV status into N- (n = 32) and N + (n = 32).

#### Statistical analysis

Data were analyzed using GraphPad Prism software version 8.4.3 (GraphPad Software, San Diego, California, USA). Normality tests revealed non-parametrically distributed women data; therefore, a Kruskal–Walli’s test and Dunn’s multiple comparison post hoc test were conducted. These results were represented as the median and interquartile range (IQR). The Hardy–Weinberg equilibrium (HWE) test was used to check for conformance to the observed frequencies of the genotypes. Frequency and percentage were used to describe the presence of the genotypes. Subgroups were compared using the Chi-squared test or Fisher’s exact test as appropriate. The strength of association was quantified using odds ratios (OR) with their corresponding 95% confidence intervals (CI) for categorical data, while numeric data were analyzed using Wilcoxon rank sum tests. A significance level of *p* < 0.05 was used. Demographic analyses utilized one-way ANOVA tests with GraphPad Prism 8.43 software (GraphPad Software, San Diego, CA, USA), and multiple comparisons were adjusted using the Bonferroni correction test.

## Results

The study consisted of 187 pregnant women; PE (n = 123; 66%) and normotensive pregnant (n = 64; 34%) women. The PE group was subdivided into EOPE (n = 64; 34%) and LOPE (n = 59; 32%) and was further stratified by HIV status EOPE− (n = 32;17%), EOPE + (n = 30; 16%), LOPE− (n = 30; 16%), and LOPE + (n = 29; 15.5%). The normotensive group was equally divided by HIV status into N− (n = 32; 17%) and N + (n = 32; 17%).

### Patient demographic

Table 1 represents women’s demographics and statistical differences across the study groups. Gestational age at delivery was significantly lower in preeclampsia compared to the normotensive (N) pregnant group (*< *0.0001****), and both systolic and diastolic blood pressure values, were significantly higher in PE compared to the normotensive group respectively (< 0.0001****). A significant difference was also observed in maternal weight between the preeclampsia and the normotensive pregnant group (0.0038**), which was significantly higher in the preeclampsia compared to the normotensive pregnant group (0.0005***).

**Table 1:** Women’s demographics and clinical data

Women data	N (n = 64)	PE (n = 123)	EOPE (n = 64)	LOPE (n = 59)	*p* value	*p* value (across all groups)
Gestational age (weeks)	38.14 (37.00–39.00)	32.70 (29.00–37.00)	28.61 (25.50–32.00)	36.80 (35.00–38.50)		< 0.0001****
N vs PE					< 0.0001****	
N− vs N +					0.0099**	
N *vs.* EOPE					< 0.0001****	
N *vs.* LOPE					< 0.0001****	
EOPE *vs.* LOPE					< 0.0001****	
Systolic BP (mmHg)	112.8 (108.0 –120.0)	158.7 (153.2–164.0)	159.4 (154.0–163.0)	158.0 (151.0–164.0)		< 0.0001****
N vs PE					< 0.0001****	
N− vs N +					0.0059^**^	
N *vs.* EOPE					< 0.0001****	
N *vs.* LOPE					< 0.0001****	
EOPE *vs.* LOPE					0.3069 ns	
Diastolic BP (mmHg)	69.58 (65.00–74.00)	94.35 (90.00–105.0)	102.0 (94.00–107.0)	99.61 (94.75–106.0)		< 0.0001****
N vs PE					< 0.0001****	
N− vs N +					0.7347 ns	
N *vs.* EOPE					< 0.0001****	
N *vs.* LOPE					< 0.0001****	
EOPE *vs.* LOPE					0.2603 ns	
Maternal weight (kg)	71.22 (60.00–80.00)	78.85 (66.00–90.00)	77.47 (68.00–90.75)	80.21 (65.00–91.25)		0.0038**
N vs PE					0.0008***	
N− vs N +					0.1461 ns	
N *vs.* EOPE					0.0054**	
N *vs.* LOPE					0.0059**	
EOPE *vs.* LOPE					> 0.9999 ns	
Maternal age (years)	26.33 (21.00–31.00)	29.19 (24.00–34.00)	30.33 (26.00–35.00)	28.06 (23.75–32.00)		0.0005***
N vs PE					0.0007***	
N− vs N +					0.0002**	
N *vs.* EOPE					0.0001***	
N *vs.* LOPE					0.0711 ns	
EOPE *vs.* LOPE					0.0475*	
Gravity	2.075 (1.00–3.00)	2.333 (1.00–3.00)	2.672 (1.00–3.00)	2.000 (1.00–3.00)		0.0929 ns
N vs PE					0.3264 ns	
N− vs N +					0.1139 ns	
N *vs.* EOPE					0.0531 ns	
N *vs.* LOPE					0.7625 ns	
EOPE *vs.* LOPE					0.0581 ns	
Parity	1.383 (0.000–2.000)	1.268 (0.000–2.000)	2.672 (1.000–3.000)	2.000 (1.000–3.000)		0.6714 ns
N vs PE					0.3738 ns	
N− vs N +					0.2748 ns	
N *vs.* EOPE					0.4370 ns	
N *vs.* LOPE					0.4915 ns	
EOPE *vs.* LOPE					0.9648 ns	

N− Normotensive HIV negative, N+ Normotensive HIV positive, EOPE− Early-onset Preeclampsia HIV negative, EOPE+ Early-onset Preeclampsia HIV positive, LOPE− Late-onset Preeclampsia HIV negative, LOPE+ Late-onset Preeclampsia HIV positive

All values are represented as median (IQR). Asterisks (*) denote significance: ∗∗*p* < 0.01 and ∗∗∗∗*p* < 0.0001. ns non-significant

### Genotyping

The association of gene polymorphisms of sFlt-1 and PlGF (rs722503, rs4903273) in preeclampsia comorbid HIV infection is tabulated below. An allelic (Table 2) and genotypic comparison of frequencies were calculated and four genetic models were tested; *i.e.* the co-dominant (equal effect of two alleles from a gene pair), dominant (alleles with the same phenotype irrespective of whether the paired allele is identical or not) [33], recessive (creates a phenotype only when the paired alleles are identical) and over the dominant model (heterozygote has a greater effect compared to the homozygote) [34]. These four genetic models were tested for associations with preeclampsia comorbid with HIV infection for each of the three variants examined (Tables 3 and 4).

**Table 2:** Genotype and allele frequency distribution (%) gene polymorphisms (rs722503 and rs 4,903,273) across pregnancy types stratified by HIV status

SNP ID		Normotensive		Preeclamptic						Pregnancy Type		HIV Status	
Rs722503 T > C		N + (n = 32)	N- (n = 32)	EOPE (n = 64)	LOPE (n = 59)	EOPE− (n = 32)	EOPE + (n = 30)	LOPE− (n = 30)	LOPE + (n = 29)	N (n = 64)	PE (n = 123)	HIV− (n = 94)	HIV + (n = 93)
Genotype co-dominant	TT	7 (21.88%)	8 (25%)	16 (25%)	13 (22.03%)	9 (28.13%)	7 (21.88%)	7 (23.33%)	6 (20.68%)	15 (23.44%)	29 (23.58%)	23 (24.47%)	21 (22.58%)
	TC	15 (46.88%)	12 (37.5%)	27 (42.19%)	19 (32.20%)	11 (34.38%)	16 (50%)	10 (33.33%)	9 (31.03%)	27 (42.19%)	46 (37.38%)	35 (37.23%)	37 (39.78%)
	CC	10 (31.23%)	12 (37.5%)	21 (32.81%)	27 (45.76%)	12 (37.5%)	9 (28.13%)	13 (43.33%)	14 (48.28%)	22 (34.37%)	48 (39.02%)	36 (38.29%)	35 (37.63%
Allele	T Major	29 (45.31%)	28 (43.75%)	59 (46.09%)	45 (38.14%)	29 (45.31%)	30 (46.88%)	24 (40%)	21 (36.21%)	57 (44.53%)	104 (42.28%)	81 (43.09%)	79 (42.47%)
	C minor	35 (54.69%)	36 (56.25%)	69 (53.91%)	73 (61.86%)	35 (54.69%)	34 (53.13%)	36 (60%)	37 (63.79%)	71 (55.47%)	142 (57.72%)	106 (56.91%)	106 (57.537%)

Rs4903273 A > G		N + (n = 32)	N− (n = 32)	EOPE (n = 64)	LOPE (n = 59)	EOPE− (n = 32)	EOPE + (n = 32)	LOPE− (n = 30)	LOPE + (n = 29)	N (N = 64)	PE (n = 123)	HIV− (n = 93)	HIV + (n = 93)
Genotype Co-dominant	AA	3 (9.38%)	4 (12.12%)	7 (10.94%)	8 (13.559%)	2 (6.25%)	5 (15.623%)	6 (20%)	2 (6.89%)	7 (11.11%)	15 (12.19%)	12 (12.90%)	10 (10.75%)
	AG	11 (34.38%)	17 (54.84%)	25 (39.06%)	22 (37.288%)	13 (40.63%)	12 (37.5%)	11 (36.67%)	11 (37.93%)	28 (44.44%)	47 (38.21%)	41 (44.09%)	34 (36.56%)
	GG	18 (56.25%)	10 (32.26%)	32 (50%)	29 (59.152%)	17 (53.13%)	15 (46.86%)	13 (43.33%)	16 (55.12%)	28 (44.44%)	61 (49.59%)	40 (43.01%)	49 (52.69%)
Allele	A major	17 (26.56%)	25 (40.32%)	49 (30.47%)	38 (32.203%)	17 (26.56%)	22 (34.38%)	23 (38.33%)	24 (25.86%)	42 (32.81%)	77 (31.30%)	65 (34.95%)	54 (29.03%)
	G minor	47 (73.437%)	37 (59.677%)	89 (69.531%)	80 (67.796%)	47 (73.437%)	42 (65.625%)	37 (61.667%)	43 (74.137%)	84 (65.625%)	169 (68.699%)	121 (65.053%)	132 (70.967%)

**Table 3:** Genotypic and allelic associations of gene polymorphisms rs722503 across study groups

SNP Rs722503 T > C Genotype		N−* vs* PE- OR (95% CI), *p*-value	N + *vs* PE OR (95% CI), *p* value	EOPE− *vs.* EOPE + OR (95% CI), *p* value	LOPE− *vs.* LOPE + OR (95% CI), *p* value	N *vs.* PE OR (95% CI), *p* value	HIV− *vs.* HIV + OR (95% CI), *p* value	N vs. EOPE OR (95% CI), *p* value	N vs. LOPE OR (95% CI), *p* value	EOPE vs. LOPE OR (95% CI), *p* value
Co-dominant	TT *vs* CC	1.042 (0.3688–3.038) *p* > 0.9999	0.7768 (0.2523–2.326) *p* = 0.7768	0.3610 (0.5625–6.978) *p* = 0.3610	1.256 (0.3071–4.187) p > 0.9999	1.129 (0.5151–2.582) *p* = 0.8382	1.065 (0.5088–2.248) *p* > 0.9999	1.151 (0.4899–2.698) *p* = 0.8202	0.9965 (0.3770 -2.678) *p* > 0.9999	1.114 (0.4274 -2.981) *p* > 0.9999
	TT vs TC	0.9583 (0.3388–2.828) *p* > 0.9999	1.346 (0.4227–4.067) *p* = 0.7630	1.714 (0.5257–6.217) *p* = 0.5325	1.050 (0.2858–4.019) *p* > 0.9999	0.8812 (0.4143–1.922) *p* = 0.8432	1.158 (0.5559–2.437) *p* = 0.8484	0.7292 (0.3054–1.728) *p* = 0.6446	1.154 (0.4834–2.823) *p* = 0.8184	0.8661 (0.3455–2.211) *p* = 0.8135
	TC vs CC	1.087 (0.3903–0.035) *p* > 0.9999	0.560 (0.2192–1.510) *p* = *0.3222*	1.091 (0.3808–3.178) *p* > 0.9999	1.197 (0.3928–3.594) *p* = > 0.9999	1.281 (0.6465–2.594) *p* = 0.5972	0.9197 (0.4673–1.803) *p* = 0.8677	1.578 (0.6912–3.399) *p* = 0.3123	0.8636 (0.3939–1.869) *p* = 0.8363	1.827 (0.7769–4.099) *p* = 0.1565
Dominant	TT vs TC + CC	1.000 (0.3680–2.575) *p* > 0.9999	0.9908 (00.3801–2.886) *p* > *0.9999*	1.789 (0.5603–5.190) *p* = 0.3951	1.623 (0.4935–5.685) *p* = 0.5389	0.9923 (0.4822–1.987) *p* > 0.9999	1.111 (0.5748–2.173) *p* = 0.8634	0.9184 (0.4176–1.995) *p* > 0.9999	1.083 (0.4662–2.612) *p* > 0.9999	1.179 (0.5208–2.807) *p* = 0.8321
Recessive	TT + TC ***vs*** CC	1.068 (0.4458–2.477) *p* = > 0.9999	0.6263 (0.2514–1.560) *p* = 0.3703	1.328 (0.5074–3.375) *p* = 0.6285	2.005 (0.7668–5.094) *p* = 0.2217	1.076 (0.5758–2.000) *p* = 0.8716	0.8884 (0.4847–1.623) *p* = 0.7617	1.227 (0.5811–2.457) *p* = 0.5868	0.7989 (0.3704–1.689) *p* = 0.6993	1.728 (0.8119–3.528) *p* = 0.1951
Over dominant	TT + CC vs TC	0.9350 (0.3851–2.184) *p* = 0.9999	1.618 (0.6457–4.214) *p* = 0.3692	1.159 (0.4483–3.139) *p* = 0.8109	0.9000 (0.2873–2.717) *p* > 0.9999	0.8187 (0.4514–1.499) *p* = 0.5318	1.114 0.6184–2.015) *p* = 0.7648	0.669 (0.3355–1.395) *p* = 0.3614	1.156 (0.5573–2.413) *p* = 0.7191	0.6509 (0.3129–1.401) *p* = 0.2694
Allele (major *vs* minor)	T *vs.* C	1.042 (0.5779–1.944) *p* > 0.9999	1.154 (0.6405–2.163) *p* = 0.7554	0.9390 (0.4678–1.879) *p* = > 0.9999	1.175 (0.5724–2.443) *p* = 0.7078	0.9572 (0.6268–1.489) *p* = 0.9117	1.038 (0.6849–1.575) *p* = 0.9168	0.9389 (0.5655–1.556) *p* = 0.9001	1.302 (0.7873–2.176) *p* = 0.3647	1.387 (0.8404–2.316) *p* = 0.2452

OR Odds ratio, CI Confidence Intervals, N− Normotensive HIV negative, N+ Normotensive HIV positive, EOPE− Early-onset Preeclampsia HIV negative, EOPE+ Early-onset Preeclampsia HIV positive, LOPE− Late-onset Preeclampsia HIV negative, LOPE+ Late-onset Preeclampsia HIV positive

Asterisks (*) denote significance: ∗*p* < 0.05 and ∗∗*p* < 0.01

**Table 4:** Genotypic and allelic associations rs4903273 (PlGF) across pregnancy types stratified by HIV status

SNP rs4903273 G > T Genotype		N− *vs.* PE− OR (95% CI), *p* value	N + vs PE + OR (95% CI), p value	EOPE− *vs.* EOPE + OR (95% CI), *p* value	LOPE− vs. LOPE + OR (95% CI), *p* value	N *vs.* PE OR (95% CI), *p* value	HIV− *vs.* HIV + OR (95% CI), *p* value	N vs. EOPE OR (95% CI), *p* value	N vs. LOPE OR (95% CI), *p* value	EOPE vs. LOPE OR (95% CI), *p* value
Codominant	AA *vs.* AG	0.7059 (0.2109–2.667) *p* = *0.7433*	0.8961 (0.2159–3.554) *p* > 0.9999	0.3692 (0.06596–2.517) *p* = 0.4025	3.000 (0.4967–16.55) *p* = 0.4069	0.8016 (0.3052–2.109) *p* = 0.8016	0.9951 (0.3822–2.512) *p* > 0.9999	0.8929 (0.2851–2.797) *p* > 0.9999	0.6875 (0.2249–2.011) *p* = 0.5668	0.7700 (0.2486–2.252) *p* = 0.7699
	AA *vs* GG	0.8333 (0.2512–3.038) *p* > 0.9999	0.7815 (0.2002–3.621) *p* > 0.9999	0.3529 (0.06459–2.209) *p* = 0.4075	3.000 (0.4967–16.55) *p* = 0.4069	1.017 (0.3959–2.684) *p* > 0.9999	1.470 (0.5839–3.957) *p* = 0.4790	1.143 (0.3743–3.487) *p* > 0.9999	0.9063 (0.3061–2.638) *p* = 0.7699	0.7700 (0.2486–2.252) *p* = 0.7699
	AG *vs* GG	1.181 (0.4992–2.795) *p* = 0.8281	0.8721 (0.3334–2.108) *p* = 0.8169	0.9559 (0.3492–2.625) *p* > 0.9999	1.231 (0.3961–3.902) *p* = 0.7816	1.298 (0.6720–2.517) *p* = 0.5090	1.477 (0.7793–2.702) *p* = 0.2725	1.280 (0.6294–2.636) *p* = 0.5738	1.318 (0.6216–2.850) *p* = 0.5617	1.030 (0.4829–2.211) *p* > 0.9999
Dominant	AA *vs* AG + GG	0.7714 (0.2440–2.478) *p* = 0.7625	0.8265 (0.2194–3.418) *p* > 0.9999	0.3600 (0.06828–1.970) *p* = 0.4258	3.375 (0.7205–17.34) *p* = 0.2542	1.400 (0.6315–1.408) *p* = 0.6014	1.537 (0.6644–3.609) *p* = 0.3926	1.583 (0.5518–4.522) *p* > 0.9999	0.7969 (0.2968–2.467) *p* = 0.7854	0.7829 (0.2918–2.421) *p* = 0.7847
Recessive	AA + AG *vs* GG	1.094 (0.4720–2.354) *p* = 0.8409	0.8510 (0.3635–2.051) *p* = 0.8260	0.7785 (0.2777–2.119) *p* = 0.8029	1.609 (0.5430–4.336) *P* = 0.4389	0.7060 (0.3945–1.286) *p* = 0.2883	1.476 (0.8422–2.618) *p* = 0.2402	1.250 (0.6240–2.534) *p* = 0.5954	1.208 (0.5846–2.522) *p* = 0.7168	0.9667 (0.4697–1.985) *p* > 0.9999
over dominant	AA + GG vs AG	0.8173 (0.3767–1.795) *p* = 0.6801	1.100 (0.4385–2.795 *p* > 0.9999	0.8769 (0.3423–2.539) *p* > 0.9999	1.056 (0.3772–2.961) > 0.9999	2.706 (1.199–5.979) *p* = 0.0234*	0.7309 (0.4034–1.307) *p* = 0.3699	2.804 (1.151–6.89) *p* = 0.0326*	2.601 (1.0310–6.539) *p* = 0.0492*	0.9276 (0.4609–1.896) *p* = 0.8549
Allele (Major *vs* minor)	*A vs* G	1.436 (0.7579–2.664) *p* = 0.3278	0.8309 (0.4289–1.641) *p* = 0.6149	0.6905 (0.3171–1.446) *p* = 0.4426	1.114 (0.5244–2.290) *p* = 0.8545	1.097 (0.6997–1.730) *p* = 0.7252	1.313 (0.8412–2.015) *p* = 0.2663	0.9082 (0.5445–1.503) *p* = 0.7956	1.053 (0.6248–1.784) *p* = 0.8919	1.159 (0.6860–1.918) *p* = 0.5988

OR odds ratio, CI confidence intervals, N− normotensive HIV negative, N+ normotensive HIV positive, EOPE− early-onset preeclampsia HIV negative, EOPE+ early-onset preeclampsia HIV positive, LOPE− late-onset preeclampsia HIV negative, LOPE+ late-onset preeclampsia HIV positive. Asterisks (*) denote significance: *p < 0.05 and **p < 0.01

### Genetic polymorphisms rs722503

#### Allelic association of rs722503 (sFlt-1) in preeclampsia comorbid HIV infection

##### Normotensive HIV−negative pregnant women vs preeclamptic HIV negative

The genotype frequencies of rs722503 in normotensive HIV−negative pregnant women were TT 7 (21.88%), TC 15 (46.88%), and CC 10 (31.23%) compared to TT 16 (25%), TC 21 (32.8%) and CC 38 (59.37%) in the preeclamptic HIV−negative group. The allele frequencies of T and C were 28 (43.75%) and 36 (56.25%) in normotensive HIV−negative pregnant and 53 (43.08%) and 71 (57.72%) in preeclamptic HIV−negative women respectively (Table 2).

The genotypic and allelic frequency associations of gene polymorphisms in normotensive HIV−negative compared to preeclamptic HIV−negative women. The genotypic frequencies of TT *vs* CC, TT *vs* TC, and TC *vs* CC showed no significant associations with OR = 1.042 (95% CI 0.3688–3.038), 0.9583 (95% CI 0.3388–2.2828), and 1.087 (95% CI 0.3903–3.035) with adjusted *p* values greater than 0.9999. Similarly, the allelic frequency association between T and C also showed no significant difference between normotensive HIV−negative compared to preeclamptic HIV−negative women with an OR of 1.042 (95% CI 0.5779–1.944) and an adjusted *p*-value greater than 0.9999. Dominant, recessive, and/or overdominant alleles (TT *vs* TC + CC; TT + TC *vs* CC and TT + CC *vs* TC) show no significant associations between normotensive HIV−negative pregnant women, with ORs of 1.000 (95% CI 0.3680–2.575); 1.068 (95% CI 0.4458–2.477); and 0.9350 (95% CI 0.3851–2.184) respectively. Notably, the adjusted *p* value greater than 0.9999, shows no significant difference (Table 3).

##### Normotensive HIV positive vs preeclamptic HIV positive

The genotype frequencies of rs722503 in the normotensive HIV−positive pregnant women were TT 8 (25%), TC 12 (37.5%), and CC 12 (37.5%) compared to TT 13 (10.57%), TC 25 (20.32%) and CC 23 (18.69%) in the preeclamptic HIV positive groups (Table 2). The allele frequencies T and C were 29 (45.31%) and 35 (54.68%) in normotensive pregnant HIV−positive and 53 (43.08%) and 71 (57.72%) in preeclamptic HIV−positive women.

The genotypic and allelic frequency associations of gene polymorphisms in normotensive pregnant HIV−positive *vs* preeclamptic HIV−positive women. The results indicated no significant associations between the genotypic frequencies of TT *vs* CC (OR = 0.7538; 95% CI 0.2523–2.326; adjusted *p* = 0.7768), TT *vs* TC (OR = 1.346; 95% CI 0.4227–4.067; adjusted *p* = 0.7630), and TC *vs* CC (OR = 0.5600; 95% CI 0.2192–1.510; adjusted *p* = 0.3222) in the two groups. Similarly, the allelic frequency association between T and C also showed no significant difference (OR = 1.154; 95% CI 0.6405–2.163; adjusted *p* = 0.7554). The dominant, recessive, and over-dominant alleles of TT *vs* TC + CC (OR = 0.9908; 95% CI 0.380–2.886; adjusted *p* > 0.9999), TT + TC *vs* CC (OR = 0.6263; 95% CI 0.2514–1.560; adjusted *p* = 0.3703), and TT + CC *vs* TC (OR = 1.618; 95% CI 0.6457–4.214; adjusted *p* = 0.3769) also show no significant associations (Table 3).

##### Normotensive pregnant vs preeclamptic groups irrespective of HIV status

We report that the genotype frequencies of rs722503 in normotensive pregnant women as TT 15(23.44%), TC 27 (42.19%), and CC 22(34.37%) compared to TT 29 (23.57%), TC 46 (37.39%) and CC 48 (39.04%) in preeclamptic women, irrespective of HIV status. The allele frequencies T and C were 57 (44.53%) and 71(55.47%) in normotensive pregnant compared to 104 (42.28%) and 142 (57.72%) in preeclamptic women, irrespective of HIV status (Table 2).

The genotypic frequency association of gene polymorphism co-dominant TT *vs* CC showed no significant association between normotensive pregnant women and the preeclamptic group [OR = 1.129; 95% Cl (0.5088–2.248) adjusted *p* = 0.838]. Similarly, the genotypic frequency of TT *vs* TC [OR = 0.8812; 95% Cl (0.4143–1.922) adjusted *p* = 0.843] and TC *vs* CC [OR = 1.281; 95% Cl (0.6465–2.594) adjusted *p* = 0.5972] showed no significant association between normotensive pregnant women compared to the preeclamptic group. Dominant, recessive, and/or overdominant alleles showed no significant association between the normotensive and preeclamptic group [(TT *vs* TC + CC adjusted *p* > 0.9999); (TT + TC *vs* CC: adjusted *p* = 0.8716) and (TT + CC *vs* TC adjusted *p* = 0.5318)]. Also, the allelic frequency association between T and C showed no significant difference between normotensive pregnant women and preeclamptic group [OR = 0.9572; 95% Cl (0.6268–1.489) adjusted *p* = 0.9117] (Table 3).

##### Early-onset preeclampsia vs late-onset preeclampsia groups irrespective of HIV status

The rs722503 genotype frequencies were TT 16 (25%), TC 27 (42.18%), and CC 21(32.81%) in EOPE and TT 13 (22.03%), TC 19 (32.20%) and CC 27 (45.76%) in LOPE women, irrespectively of HIV status. The allele frequencies T and C were 59 (46.09%) and 69(53.91%) in EOPE and 45 (38.14%) and 73(61.86%) in LOPE groups irrespective of HIV status (Table 2).

The genotypic frequency association of gene polymorphism co-dominant TT *vs* CC showed no significant association of EOPE compared to LOPE women irrespective of HIV status [OR = 1.114; 95% Cl (0.4274 -2.981) adjusted *p* > 0.9999]. Similarly, the genotypic frequency was TT *vs* TC [OR = 0.8661; 95% Cl (0.3455–2.211) adjusted *p* = 0.8135] and TC *vs* CC [OR = 1.827; 95% Cl (0.7769–4.099) adjusted *p* = 0.1565] showed no significant association with EOPE compared to LOPE group. Dominant, recessive, and/or overdominant alleles showed no significant association between EOPE *vs* LOPE group [TT *vs* TC + CC adjusted *p* = 0.832; TT + TC *vs* CC: adjusted *p* = 0.195 and TT + CC *vs* TC adjusted *p* = 0.2694]. Also, the allelic frequency association T compared C showed no significant difference between EOPE and LOPE groups [OR = 1.387; 95% Cl (0.8404–2.316) adjusted *p* = 0.2452] (Table 3).

##### Normotensive vs early-onset preeclampsia groups irrespective of HIV status

The rs722503 genotype frequencies were TT 15 (23.44%), TC 27 (42.19%), and CC 22 (34.37%) in normotensive pregnant and TT 16 (25%), TC 27 (42.18%) and CC 21 (32.8%) in EOPE women, irrespectively of HIV status. The allele frequencies T and C were 57 (44.53%) and 71 (55.47%) in normotensive pregnant women and 59 (46.09%) and 69 (53.91%) in EOPE women irrespective of HIV status (Table 2).

The genotypic frequency association of gene polymorphism codominant TT *vs* CC showed no significant association of normotensive pregnant women compared to the EOPE group [OR = 1.151 95% Cl (0.4899–2.698); adjusted *p* = 0.8202]. Also, the genotypic frequency of TT *vs* TC [OR = 0.7292; 95% Cl (0.3054–1.728) adjusted *p* = 0.6446] and TC *vs* CC [OR = 1.578; 95% Cl (0.6912–3.399) adjusted *p* = 0.3123] showed no significant association with normotensive pregnant women compared to EOPE women. Dominant, recessive, and/or overdominant alleles showed no significant association between normotensive pregnant women compared to the EOPE group [TT *vs* TC + CC adjusted *p* > 0.9999; TT + TC *vs* CC: adjusted *p* = 0.5868 and TT + CC *vs* TC Adjusted *p* = 0.3614]. Also, the allelic frequency association T *vs* C showed no significant difference between normotensive pregnant women and the EOPE group [OR = 0.9389; 95% Cl (0.5655–1.556) adjusted *p* = 0.9001] (Table 3).

##### Normotensive vs late-onset preeclampsia women irrespective of HIV status

The rs722503 genotype frequencies of TT 15 (23.44%), TC 27 (42.19%), and CC 22 (34.37%) in normotensive pregnant women and 13 (22.03%), 19 (32.20%) and 27 (45.76%) in LOPE group, respectively of HIV status. The allele frequencies T and C were 57 (44.053%) and 71 (55.47%) in normotensive pregnant women and 45 (38.14%) and 73 (61.86%) in the LOPE group irrespective of HIV status (Table 2).

The genotypic frequency association of gene polymorphism codominant TT *vs* CC showed no significant association of normotensive pregnant women with the LOPE group irrespective of HIV status [OR = 0.9965%; 95% Cl (0.3770–2.678); adjusted *p* > 0.9999]. Also, the genotypic frequency of TT *vs* TC [OR = 1.154; 95% Cl (0.4834–2.823); adjusted *p* = 0.8184] and TC *vs* CC [OR = 0.8636; 95% Cl (0.3939–1.869); *p* = 0.8363] showed no significant association with normotensive compared to LOPE groups. Dominant, recessive, over dominant alleles showed no significant association between normotensive pregnant women *vs* LOPE group [TT *vs* TC + CC adjusted *p* > 0.9999; TT + TC *vs* CC: adjusted *p* = 0.6993 and TT + CC *vs* TC adjusted *p* = 0.7191]. Also, the allelic frequency association T *vs* C showed no significant difference between normotensive pregnant women and the LOPE group [OR = 1.302; 95% Cl (0.7873–2.176); adjusted *p* = 0.3647] (Table 3).

##### HIV negative vs HIV−positive women irrespective of pregnancy type

The rs722503 genotype frequencies of TT 23 (24.47%), TC 35 (37.38%), and CC 36 (38.29%) in HIV−negative pregnant women compared to TT 21 (22.58%), TC 37 (39.78%), and CC 35 (37.63%) in HIV−positive pregnant women, irrespective of pregnancy type. The allele frequencies T and C were 81 (43.09%) and 106 (59.91%) in the HIV−negative pregnant women and 79 (42.47%) compared to 107 (57.53%) in HIV−positive pregnant women irrespective of pregnancy type (Table 2).

The genotypic frequency association of gene polymorphism codominant TT *vs* CC, TT *vs* TC, and TC *vs* CC showed no significant association, in HIV−negative compared to HIV−positive pregnant women. Additionally, dominant, recessive, over dominant alleles showed no significant association between HIV− *vs* HIV + group [TT *vs* TC + CC adjusted *p* = 0.8634; TT + TC *vs* CC: adjusted *p* = 0.7617 and TT + CC *vs* TC adjusted *p* = 0.7648]. Also, the allelic frequency association T *vs* C showed no significant difference between the HIV− and HIV + group [OR = 1.038; 95% Cl (0.6849–1.575); adjusted *p* = 0.9168] (Table 3).

### Genetic polymorphisms rs4903273

#### Normotensive HIV−negative pregnant vs preeclamptic HIV− negative women

The genotype frequencies of rs493273 AA 4 (12.12%), AG 17 (54.84%), and GG 10 (32.26%) in normotensive HIV−negative pregnant women and AA 8 (12.5%), AG 24 (37.5%) and GG 30 (46.87%) in preeclamptic HIV−negative (Table 2). The allele frequencies A and G were 25 (39.06%) and 37 (57.81%) in normotensive women HIV−negative pregnant compared to 40 (32.52%) and 84 (68.29%) in preeclamptic HIV−negative women groups.

The genotypic and allelic frequency associations of gene polymorphism in normotensive HIV−negative women compared to preeclamptic HIV−negative women. The result shows no significant association between the genotypic frequencies of AA *vs* GG [OR = 0.8333; 95% CI 0.2512–3.038; adjusted *p* > 0.9999], AA *vs* AG [OR = 0.7433; 95% CI (0.7059–2.667); adjusted *p* = 0.7433], and AG *vs* GG [OR = 1.181; 95% CI (0.4992–2.795); adjusted *p* = 0.8281] in the two groups. Similarly, when considering dominant, recessive, and over-dominant alleles, no significant associations were found between normotensive HIV−negative pregnant and preeclamptic HIV−negative groups. The comparisons of AA *vs* AG + GG [OR = 0.7714; 95% CI (0.2440–2.478); adjusted *p* = 0.7625], AA + AG *vs* GG [OR = 1.094; 95% CI (0.4720–2.354); adjusted *p* = 0.8409), and AA + GG *vs* AG [OR = 0.8173; 95% CI (0.3767–1.798); adjusted *p* = 0.6801] did not show significant differences. Additionally, the allelic frequency association between A and G also showed no significant difference between the two groups [OR = 1.436; 95% CI (0.7579–2.664); adjusted* p* = 0.3278] (Table 3).

#### Normotensive HIV positive vs preeclamptic HIV positive

The genotype frequencies of rs493273: AA 8(25%), AG 12(37.5%), and GG 12(37.5%) in normotensive HIV−positive pregnant women and AA 13(10.57%), AG 25 (20.32%) and GG 23 (18.69%) in preeclamptic HIV−positive (Table 2). The allele frequencies A and G were 17 (26.56%) and 47(73.43%) in normotensive-HIV positive pregnant women and 37 (33.33%) and 85 (69.10%) in preeclamptic-HIV positive.

The genotypic and allelic frequency associations of gene polymorphism in normotensive HIV−positive women compared to preeclamptic HIV−positive women. The study shows no significant associations between the genotypic frequencies of AA *vs* GG (OR = 0.7815; 95% CI 0.2002–3.621; adjusted *p* > 0.9999), AA vs AG (OR = 0.8961; 95% CI 0.2195–3.554; adjusted *p* > 0.9999), and AG *vs* GG (OR = 0.8721; 95% CI (0.3334–2.108); adjusted *p* = 0.8169) in the two groups. Additionally, dominant, recessive, and over-dominant alleles did not show a significant association between the two groups, AA *vs* AG + GG OR = 0.8265 (95% CI 0.2194–3.418) with an adjusted *p* value greater than 0.9999, indicating no statistical significance. Similarly, the comparisons of AA + AG *vs* GG resulted in an OR = 0.8510 (95% CI (0.3635–2.051) with an adjusted *p* value of 0.8260, and AA + GG *vs* AG showed an OR = 1.100 (95% CI 0.4385–2.725) with an adjusted *p* value greater than 0.9999, all indicating no significant associations. Furthermore, the allelic frequency association between A and G also showed no significant difference between the two groups (OR = 0.8309; 95% CI (0.4289–1.641); adjusted *p* = 0.6149 (Table 3).

#### Normotensive vs preeclamptic groups irrespective of HIV status

The rs4903273 genotype frequencies in normotensive pregnant women were AA 7 (11.11%), AG 28 (44.44%) and GG 28 (34.44%) compared to AA 15 (12.19%), AG 47 (38.21%), and GG 61 (49.59%) in preeclamptic women, irrespective of HIV status. The allele frequencies A and G were 42(32.81%) and 84 (65.63%) in normotensive pregnant women compared to 77 (31.30%) and 169 (68.69%) in preeclamptic women, irrespective of HIV status (Table 2).

The genotypic frequency association of gene polymorphism co-dominant AA *vs* GG showed no significant association between the normotensive pregnant compared to preeclamptic groups [OR = 1.017; 95% Cl (0.3959–2.684); adjusted *p* > 0.9999]. Similarly, the genotypic frequency of AA *vs* AG [OR = 0.8016; 95% Cl (0.3052–2.109); adjusted *p* = 0.8016] and AG *vs* GG [OR = 1.298; 95% Cl (0.6720–2.517); adjusted *p* = 0.5090] showed no significant association with normotensive pregnant women compared to preeclamptic groups. Dominant and recessive alleles showed no significant association between the normotensive pregnant women *vs* the preeclamptic group [AA *vs* AG + GG adjusted *p* = 0.6014; AA + AG *vs* GG: adjusted *p* = 0.2883]. However, over dominant alleles AA + GG *vs* AG showed a significant difference [OR = 2.706; 95% Cl (1.199–5.979); adjusted *p* = 0.0234*]. Also, the allelic frequency association A *vs* G showed no significant difference between normotensive pregnant women and the preeclamptic group [OR = 1.097; 95% Cl (0.6997–1.730); adjusted *p* = 0.7252] (Table 4).

#### Early-onset preeclampsia vs late-onset preeclampsia groups irrespective of HIV status

The rs4903273 genotype frequencies of AA 7(10.94%), AG 25 (39.06%) and GG 32 (50%) in EOPE and AA 8 (13.56%), AG 22 (37.29%) and GG 29 (59.15%) in LOPE women, irrespective of HIV status. The allele frequencies A and G were 49 (30.47%) and 89(69.53%) in EOPE women compared to 38 (32.20%) and 80 (67.79%) in LOPE women irrespective of HIV status (Table 2).

The genotypic frequency association of gene polymorphism codominant AA *vs* GG showed no significant association of EOPE with the LOPE group [OR = 0.7700; 95% Cl (0.2486–2.252); adjusted *p* = 0.7699]. Similarly, the genotypic frequency of AA *vs* AG [OR = 0.7700; 95%Cl (0.2486–2.252); adjusted *p* = 0.7699] and AG *vs* GG [OR = 1.030; 95% Cl (0.4829–2.211); adjusted *p* > 0.9999] showed no significant association with EOPE compared to LOPE groups. Dominant, recessive, over dominant alleles showed no significant association between EOPE *vs* LOPE group [AA *vs* AG + GG; adjusted *p* = 0.7847; AA + AG *vs* GG; adjusted *p* > 0.9999 and AA + GG *vs* AG; adjusted *p* = 0.8549]. Also, the allelic frequency association A *vs* G showed no significant difference between the EOPE and LOPE group [OR = 1.159; 95% Cl (0.6860–1.918); adjusted *p* = 0.5988] (Table 4).

#### Normotensive vs early-onset preeclampsia group irrespective of HIV status

The rs4903273 genotype frequencies of AA 7 (11.11%), AG 28 (44.44%) and GG 28 (34.44%) in normotensive pregnant compared to AA 7 (10.943%), AG 25 (39.06%) and GG 32 (50%) in EOPE groups, irrespective of HIV status. The allele frequencies A and G were 42 (32.81%) and 84 (65.63%) in normotensive pregnant compared to 49 (30.47%) and 89 (69.53%) in EOPE groups, irrespective of HIV status (Table 2).

The genotypic frequency association of gene polymorphism co-dominant AA *vs* GG showed no significant association of normotensive pregnant women with the EOPE group [OR = 1.143; 95% Cl (0.3743–3.487); adjusted *p* > 0.9999]. Also, the genotypic frequency of AA *vs* AG [OR = 0.8929; 95% Cl (0.2851–2.797); adjusted *p* > 0.9999)] and AG *vs* GG [OR = 1.280; 95%Cl (0.6294–2.636); adjusted *p* = 0.5738] showed no significant association with normotensive pregnant compared to EOPE groups. Dominant and recessive alleles showed no significant association between the normotensive pregnant *vs* EOPE group [AA *vs* AG + GG adjusted *p* > 0.9999; AA + AG *vs* GG: adjusted *p* = 0.5954]. In contrast to over-dominant alleles AA + GG *vs* AG showed significant differences between normotensive pregnant compared to EOPE groups [OR = 2.804; 95% Cl (1.151–6.89); adjusted *p* = 0.0326*]. The allelic frequency association A *vs* G showed no significant difference between normotensive pregnant women and the EOPE group [OR = 0.9082; 95% Cl (0.5445–1.503); adjusted *p* = 0.7956] (Table 4).

#### Normotensive *vs* late-onset preeclampsia groups irrespective of HIV status

Table 2, the rs4903273 genotype frequencies in the normotensive pregnant group were AA 7 (11.11%), AG 28 (44.44%), and GG 28 (34.44%) compared to 8 (13.56%), 22 (37.29%) and 29 (59.15%) in LOPE group, irrespective of HIV status. The allele frequencies A and G were 42 (32.81%) and 84 (65.63%) in normotensive pregnant compared to 38 (32.20%) and 80 (67.79%) in the LOPE groups, irrespective of HIV status (Table 2).

The genotypic frequency association of gene polymorphism codominant AA *vs* GG showed no significant association of normotensive pregnant women with the LOPE group [OR = 0.9063% Cl (0.3061–2.638); adjusted *p* = 0.7699]. Also, the genotypic frequency of AA *vs* AG [OR = 0.6875; 95% Cl (0.2249–2.011); adjusted *p* = 0.5668] and AG *vs* GG [OR = 1.318; 95% Cl (0.6216–2.850); adjusted *p* = 0.5617] showed no significant association with normotensive pregnant women compared to EOPE groups. Dominant and recessive alleles showed no significant association between normotensive pregnant women *vs* the LOPE group [AA *vs* AG + GG; adjusted *p* = 0.7854 and AA + AG *vs*. GG; adjusted *p* = 0.7168]. However, over dominant alleles AA + AG *vs* GG showed a significant difference between normotensive pregnant compared LOPE groups [OR = 2.601; 95% Cl (1.031–6.539); adjusted *p* = 0.0492*] Also, the allelic frequency association A *vs* G showed no significant difference between normotensive pregnant women and LOPE groups irrespective of HIV status [OR = 1.053; 95% Cl (0.6248–1.784); adjusted *p* = 0.8919] (Table 4).

#### HIV negative vs HIV positive groups irrespective of pregnancy type

The rs4903273 genotype frequencies of AA 12 (12.90%), AG 41 (44.09%) and GG 40 (43.01%) in HIV−negative pregnant compared to AA 10 (10.75%), AG 34 (36.59%) and GG 49 (52.69%) in HIV−positive pregnant groups, regardless of pregnancy type. The allele frequencies A and G were 65 (34.95%) and 121 (65.05%) in HIV−negative pregnant women and 54 (29.03%) and 132 (70.97%) in HIV−positive groups, irrespective of pregnancy type (Table 2).

The genotypic frequency association of gene polymorphism co-dominant AA *vs* GG, AA vs AG and AG *vs* GG showed no significant association between HIV−negative compared to HIV−positive pregnant women. The dominant, recessive, and/or overdominant alleles showed no significant association between HIV−negative compared to HIV−positive pregnant women group [AA *vs* AG + GG; adjusted *p* = 0.3926; AA + AG *vs* GG: adjusted *p* = 0.2402 and AA + GG *vs* AG adjusted *p* = 0.3699]. Also, the allelic frequency association, A *vs* G showed no significant difference between HIV−negative and HIV−positive pregnant groups [OR = 1.313; 95% Cl (0.8412–2.015); adjusted *p* = 0.2663] (Table 4).

## Discussion

This study examined the genotypic frequencies of rs722503 variants (TT, TC, CC) in normotensive pregnant HIV−negative, normotensive pregnant HIV−positive, preeclamptic HIV−positive, and preeclamptic HIV−negative women. The analysis revealed no significant associations between normotensive and preeclampsia risk in HIV−negative pregnant women. The odds ratios (OR) for TT *vs* CC, TT *vs* TC, and TC *vs* CC were 1.042 (95% CI 0.3688–3.038), 0.9583 (95% CI 0.3388–2.2828), and 1.087 (95% CI 0.3903–3.035), respectively, all with adjusted *p*-values greater than 0.9999. Similarly, the allelic frequency association between T and C alleles showed no significant disparity between normotensive and preeclamptic groups, with an OR of 1.042 (95% CI 0.5779–1.944). Dominant, recessive, and over-dominant allele models (TT *vs* TC + CC; TT + TC *vs* CC; TT + CC *vs* TC) also did not show a significant association, with ORs of 1.000 (95% CI 0.3680–2.575), 1.068 (95% CI 0.4458–2.477), and 0.9350 (95% CI 0.3851–2.184). In normotensive HIV−positive compared to preeclamptic HIV−positive groups, the genotype frequencies analysis demonstrates no significant associations between genotypic frequencies when comparing TT *vs* CC (OR = 0.7538; 95% CI 0.2523–2.326; adjusted *p* = 0.7768), TT *vs* TC (OR = 1.346; 95% CI 0.4227–4.067; adjusted *p* = 0.7630), and TC *vs* CC (OR = 0.5600; 95% CI 0.2192–1.510; adjusted *p* = 0.3222) in the two groups. Additionally, the allelic frequency association between T and C also showed no significant difference (OR = 1.154; 95% CI 0.6405–2.163; adjusted *p* = 0.7554). Also, further analysis using dominant, recessive, and over-dominant allele models did not reveal significant associations. The TT *vs* TC + CC (OR = 0.9908; 95% CI 0.380–2.886; adjusted *p* > 0.9999), TT + TC *vs* CC (OR = 0.6263; 95% CI 0.2514–1.560; adjusted *p* = 0.3703), and TT + CC *vs* TC (OR = 1.618; 95% CI 0.6457–4.214; adjusted *p* = 0.3769) showed no significant differences. These findings suggest that the rs722503 polymorphism is not significantly associated with an increased risk of preeclampsia development in HIV−positive pregnant women of African ancestry. Furthermore, this study reports that preeclamptic individuals had a higher homozygous (TT and CC) genotypes; a heterozygous disadvantage since the heterozygous genotype and the TC alleles were higher in normotensive compared to preeclamptic groups irrespective of HIV status. Statistical analysis of the dominant, recessive, and over-dominant allele frequency associations also showed no significant associations with preeclampsia.

In contrast, other studies have reported associations between rs722503 variants and preeclampsia risk in different populations [32, 35, 36]. The rs722503 has been reported to be associated with an increased risk of PE development in Iranian and Caucasian pregnant women [35]. Their results are also corroborated by the findings of Valenzuela (2012) and Amosco (2016) who also noted that rs722503 polymorphisms are associated with PE predisposition in caucasian and Filipino women but not in Black women [32, 36]. Additionally, the data analyzed by Srinivas SK et al. show an association between rs722503 polymorphism and preeclampsia incidence in white populations, the T allele was associated with a higher risk of preeclampsia (OR 2.12, 95% CI 1.07–4.19, *p* = 0.03) [37]. These findings suggest that discrepancies in human ethnicity and HIV status may play a crucial role in the varying conclusions drawn from current studies regarding the association of rs722503 with preeclampsia comorbid HIV infection**.** Thus, rs722503 polymorphism may not be responsible for the development of preeclampsia in Black South African women. The VEGF family is important for establishing normal pregnancy [38], and related rs722503 has been implicated in abnormal placentation and preeclampsia development in the white population [32]. The rs722503 polymorphism is located in intron 10 of the sFlt-1gene and can alter the regulatory motif for binding of nuclear factor-κB (NF-κB) [39, 40]. NF-κB is a transcription factor that can participate in both activation and repression of transcription and hence would influence endothelial cell proliferation [39, 40]. The effect of sFlt-1 gene polymorphisms is often studied in preeclampsia [39]. Soluble Flt-1 is encoded by an alternatively spliced transcript of sFlt-1 is an antagonist of VEGF and PIGF and is up-regulated in preeclampsia [39, 41]. This variant occurs in an intron and its mechanism of action remains unknown [32]. Since non-coding introns are important during the splicing process to produce mature mRNAs; the splicing process may be constitutive or highly tissue-specific relying on both canonical sequences at the exon–intron junction and other sequence motifs occurring throughout the transcript [32]. Mutations at the splice sites may be akin to nonsense or missense mutations, resulting in aberrantly included introns or skipped exons [42]. Notably, VEGFR-1 is alternatively spliced to generate the full-length transmembrane receptor and the soluble isoform [32].

### Placental growth factor (PlGF) rs 49,032,173

Our study investigated the genotypic and allelic frequencies of rs493273 in normotensive, preeclamptic HIV−negative pregnant women, and normotensive and preeclamptic HIV−positive pregnant women. In normotensive HIV−negative compared to preeclamptic HIV−negative, we demonstrated no significant associations in genotypic frequencies of AA *vs* GG [OR = 0.8333; 95% CI (0.2512–3.038) adjusted *p* > 0.9999; AA *vs* AG (OR = 0.7433; 95% CI (0.7059–2.667) adjusted *p* = 0.7433], and AG *vs* GG [OR = 1.181; 95% CI (0.4992–2.795); adjusted *p* = 0.8281]. Dominant, recessive, and over-dominant allele comparisons also did show significant associations between normotensive and preeclamptic HIV−negative groups [AA *vs* AG + GG (OR = 0.7714; 95% CI (0.2440–2.478); adjusted *p* = 0.7625); AA + AG vs GG [OR = 1.094; 95% CI (0.4720–2.354); adjusted *p* = 0.8409], and AA + GG vs AG [OR = 0.8173; 95% CI 0.3767–1.798; adjusted *p* = 0.6801]. Similarly, in normotensive and preeclamptic HIV−positive women, no significant associations were observed in genotypic frequencies of AA *vs* GG (OR = 0.7815; 95% CI 0.2002–3.621; adjusted *p* > 0.9999), AA *vs* AG (OR = 0.8961; 95% CI 0.2195–3.554; adjusted *p* > 0.9999), and AG *vs* GG (OR = 0.8721; 95% CI 0.3334–2.108; adjusted p = 0.8169). The dominant, recessive, and over-dominant alleles in the study show no significant associations between the normotensive and preeclamptic HIV−positive pregnant women, odds ratio OR = 0.8265 (95% CI 0.2194–3.418) with an adjusted *p* > 0.9999 for AA *vs* AG + GG, indicating no statistical significance. Similarly, the comparison of AA + AG *vs* GG resulted in an OR = 0.8510 (95% CI 0.3635–2.051) with an adjusted *p* value of 0.8260. Moreover, the comparison of AA + GG *vs* AG showed an OR = 1.100 (95% CI 0.4385–2.725) with an adjusted *p* > 0.9999. These results collectively indicate no significant associations between these allele combinations and the risk of preeclampsia in HIV−positive pregnant women of African ancestry. Additionally, the allelic frequency association between A and G also showed no significant difference between the two groups, with an OR = 0.8309 (95% CI 0.4289–1.641) and an adjusted *p*-value of 0.6149. These findings suggest that the rs493273 polymorphism is not significantly associated with the risk of preeclampsia in either HIV−negative or HIV−positive pregnant women of African ancestry, highlighting the complexity of genetic factors contributing to preeclampsia across different populations and HIV infection.

We also report differences in the rs4903273 genotype of the PLGF gene together with allele frequency variation between normotensive and preeclamptic women, irrespective of their HIV status. Specifically, the frequencies of over dominant alleles AA + GG *vs* AG showed a significant difference [OR = 2.706; 95% Cl (1.199–5.979); Adjusted *p* = 0.0234*], a previous study reported that PlGF levels were significantly reduced in women with preeclampsia compared to normotensive pregnant women [43]. This suggests a potential role of PlGF in the pathogenesis of preeclampsia. In our study, the rs4903273 genotype frequencies showed variations between normotensive and preeclamptic women, irrespective of their HIV status. Akolekar et al. highlighted the association between PlGF gene polymorphisms and susceptibility to preeclampsia, emphasizing the importance of genetic factors in the development of this preeclampsia [44]. Our study also noted a variation in the frequencies of the AA, AG, and GG genotypes, as well as the A and G alleles, between the EOPE compared to LOPE groups, irrespective of HIV status. Interestingly, the frequency of the GG genotype was higher in LOPE compared to EOPE women, suggesting that individuals with this variant may be predisposed to LOPE development. Furthermore, the frequency of the A and G alleles differed between the two groups, with the G allele being more frequent. These results suggest that the G allele may be associated with a higher risk of preeclampsia predisposition. The differential allelic expression reflects the complexity of preeclampsia pathogenesis and type as well as the potential influence of population-specific genetic variation and environmental factors. Our findings support the potential role of rs4903273 in preeclampsia development. This polymorphism is located in the intron region of the leptin receptor gene which is involved in the regulation of vascular function, angiogenesis, and inflammation, processes and is dysregulated in preeclampsia [45]. Notably, the GG genotype of rs4903273 increases the production of leptin inducing endothelial dysfunction and predisposing the development of preeclampsia [46]. In our study, the frequency of the AA genotype was slightly higher in normotensive (11.111%) than in EOPE women (10.937%), whilst the frequency of the AG genotype was more common in both groups (44.444% *vs* 39.063%). Notably, the GG genotype was more prevalent in EOPE (50%) than in normotensive (34.444%) women. These significant differences that was noted in AA + GG *vs* AG between normotensive pregnant compared to EOPE groups [OR = 2.804; 95% Cl (1.151–6.89); adjusted *p* = 0.0326*]. This significant difference could indicate a possible association between the rs4903273 variant and the susceptibility to EOPE compared to normotensive pregnant in African women. Allele frequencies of A and G differed between normotensive and EOPE women, with the latter group having a higher frequency of the G allele (69.531% *vs* 65.625%). The frequencies of genotypes, AA, AG, and GG did not differ significantly between normotensive and LOPE women, irrespective of their HIV status. However, there was a trend of higher frequencies of the G allele and the GG and AG genotypes in the LOPE group, suggesting a potential association between these genetic variations and preeclampsia development in our Black population. over dominant alleles AA + AG *vs* GG showed a significant difference between normotensive pregnant compared LOPE groups [OR = 2.601; 95% Cl (1.031–6.539); adjusted *p* = 0.0492*]. Previous studies reported that it is unusual to find an association between a disease with a heterozygous genotype nonetheless [34] reported this heterozygous genotype is protective against tuberculosis [34]. Currently, there are no studies on heterozygous genotype associated with preeclampsia development, however, in our study individuals with AG may have an impact on PE development in women of African ancestry. However, an exploration of the potential gene-environment interplay and other candidate genetic variations will advance our understanding of the pathophysiology underlying preeclampsia in women of African ancestry compared to other ethnic groups. Our study shows no significant genotypic frequency association between HIV−negative and HIV−positive pregnant women for AA *vs* GG (adjusted *p* = 0.3926), AA *vs* AG (adjusted *p* = 0.2402), and AG *vs* GG (adjusted *p* = 0.3699). Additionally, the allelic frequency association (A *vs* G) showed no significant difference between the two groups [OR = 1.313; 95% CI (0.8412–2.015); adjusted* p* = 0.2663]. These findings suggest a lack of genetic influence from the rs4903273 polymorphism on HIV susceptibility during pregnancy.

We also reported lower gestational age at delivery in the preeclampsia women compared to the Normotensive suggesting that preeclampsia may occur earlier in pregnancy in these women. The significantly higher systolic and diastolic blood pressure values in the preeclampsia women compared to the normotensive indicate the severity of the condition in these women. Maternal weight was significantly different between the preeclampsia women and the normotensive, which also affects the management of the condition (Table 1).

## Conclusion

We found no significant association between the rs722503 polymorphism and the risk of preeclampsia in HIV−positive pregnant women of African ancestry. These findings suggest that rs722503 may not play a significant role in preeclampsia development in Black South African women. In contrast, the rs4903273 gene polymorphism is not a major risk factor for preeclampsia, despite the AA + AG vs GG genotype conferring an increased risk of preeclampsia compared to normotensive pregnant women irrespective of HIV status. Also, over dominant alleles AA + AG *vs* GG showed a significant difference between normotensive pregnant compared LOPE groups [OR = 2.601; 95% Cl (1.031–6.539); adjusted *p* = 0.0492*]. However, further studies are needed to confirm these findings and to elucidate the exact mechanism by which the rs4903273 gene polymorphism may contribute to the pathogenesis of LOPE. Indeed, the genetics of preeclampsia are multifactorial, making it challenging to identify precise genetic biomarkers with clinical utility. Nevertheless, our results contribute to a better understanding of ethnic-specific risk factors for preeclampsia development whilst minimizing potential confounders such as HIV status.

## Data Availability

Not applicable.
